# Deconstructing the Native Speaker: Further Evidence From Heritage Speakers for Why This Horse Should Be Dead!

**DOI:** 10.3389/fpsyg.2021.717352

**Published:** 2021-10-05

**Authors:** Wintai Tsehaye, Tatiana Pashkova, Rosemarie Tracy, Shanley E. M. Allen

**Affiliations:** ^1^Department of English Linguistics, University of Mannheim, Mannheim, Germany; ^2^Center for Cognitive Science, University of Kaiserslautern, Kaiserslautern, Germany

**Keywords:** native speakers, heritage speakers, subordinate clauses, heritage German, majority language

## Abstract

The category “native speaker” is flawed because it fails to consider the diversity between the speaker groups falling under its scope, as highlighted in previous literature. This paper provides further evidence by focusing on the similarities and differences between heritage speakers (HSs) and monolingually-raised speakers (MSs) of their heritage and majority languages. HSs are bilinguals who acquire a family (heritage) language and a societal (majority) language in early childhood. Naturalistic exposure from early childhood qualifies them as native speakers of their heritage language. Some HSs are simultaneous bilinguals, which makes them native speakers of their majority language as well. Others are early second language acquirers who may be indistinguishable from simultaneous bilinguals. Previous research shows that the heritage language productions of German HSs in the United States do not completely overlap with those of German MSs, who are, by default, native speakers. In overall clause type selection (independent main, coordinate main, and subordinate), the HSs differ from German MSs in German but are similar to English MSs in English. The present study examines the distribution of finite subordinate clauses and their types (relative, complement, and adverbial) across registers in 27 adolescent HSs of German in the United States, compared to 32 adolescent MSs of German and 32 MSs of English. All participants described a short video in two settings (formal/informal) and two modes (spoken/written). Results demonstrate that, even with respect to a specific phenomenon (subordinate clauses), HSs show similarities and differences to MSs of both languages. Concerning the distribution of subordinate clause types, HSs behave similarly to both English and German MSs. Concerning subordinate clauses in general, HSs use them less frequently than MSs in German. In English, the difference is more nuanced: HSs differentiate between settings in both modes, while MSs do so only in the written mode. This indicates that the category “native speaker” is not a meaningful descriptor since it covers speakers with varying production patterns. We propose that studies including native speakers should assure transparency and replicability of research by specifying and taking into account speaker characteristics such as bilingualism, proficiency, exposure and dominance.

## Introduction

The category “native speaker” has been used to characterize a particular speaker population for many years (see Hopp, 2016; Azar et al., 2019; Ionin et al., 2021; Redl et al., 2021 as recent cases in point). What most researchers seem to agree on is that a native speaker is defined as a speaker who acquires their language naturalistically in early childhood (Cook, 1999; Davies, 2004, 2013). Despite its popularity, this definition can be questioned. It has been criticized for being a political and ideological construct (Bonfiglio, 2010; Dewaele, 2018) and for discrediting late second language (L2) speakers as “deficient versions of natives” (Cook, 2016, p. 186). Another point of criticism is that the category is underspecified because it does not reflect the variation within the subgroups under its scope (Davies, 2004; Lowe, 2020). This criticism holds for the specific native speaker population considered in the present study, namely heritage speakers (HSs). They are broadly defined as “bilinguals who have acquired a family (heritage language) and a majority societal language naturalistically in early childhood” (Pascual et al., 2012, p. 450). Therefore, they are native speakers of both of their languages (Montrul, 2016; Kupisch and Rothman, 2018) irrespective of them being simultaneous bilinguals or early L2 acquirers of the majority language (Rothman and Treffers-Daller, 2014, p. 96).

Comparisons of HSs with monolingually-raised speakers (MSs) reveal areas of difference and similarity (Montrul, 2016, p. 208). The similarities with MSs can be found in both their heritage language (Nagy, 2015; Nagy and Lo, 2019; Łyskawa and Nagy, 2020) and their majority language (Kupisch et al., 2014; Pashkova et al., in press). The differences also become apparent in both their languages (Rothman, 2007; Polinsky, 2018; Scontras et al., 2018 for the heritage language; Scontras et al., 2017; Polinsky, 2018; Paradis, 2019 for the majority language). It is important to mention that the differences are not clear-cut but rather gradient. For example, in a study on clause-type use across registers, we found that German HSs with majority English showed similar distributional patterns in their heritage German productions in independent main clauses and different patterns in coordinate main clauses and subordinate clauses, compared to German MSs (Pashkova et al., in press). These results illustrate a more nuanced difference in clause type productions of MSs and HSs in their heritage language. Taken together, these findings indicate that the category “native speaker” fails to adequately reflect the variation between the speaker groups who fall under its scope, in this case, HSs and MSs.

Consequently, if a linguistic study states that it examined a group of native speakers, we cannot be absolutely certain who these speakers were and if their individual patterns of language use were comparable. The native speaker group could comprise for example MSs, HSs, or late L2 acquirers who emigrated and whose first language (L1) is undergoing attrition. Unquestionably, these speakers use their native language differently. Thus, further specification of the category “native speaker” is necessary to ensure transparency and replicability of research.

In the current study, we continue to address similarities and differences between two groups of native speakers, namely HSs and MSs. Focusing on finite subordinate clauses (SCs), we investigate their general use and the use of their types (complement, adverbial, and relative) across registers. This structural spectrum offers a promising area of variation in the two native speaker sub-groups because it is located at the interface of syntax and discourse (Sorace, 2011).

On the syntactic level, mastery of SCs is a potential source of variation in heritage language due to the complexity of SCs and different word order constraints in SCs in HSs’ heritage and majority language (Pashkova et al., in press). Regarding SC types, differences in acquisition timing, paths, and the language input may play a key role in their later production (Andreou et al., 2020a). Researchers have suggested different acquisition trajectories of subordinate clause types (Vasilyeva et al., 2008; Paradis et al., 2017). In heritage language contexts, HSs and MSs presumably have similar acquisition conditions during infancy and early childhood, which then start to diverge once exposure to the majority language increases (around preschool/kindergarten), and especially once formal schooling sets in. Hence, for the heritage language, we can expect that the earliest acquired SC types will be similar in HSs’ and MSs’ productions, while the later acquired types might show more variation. In the majority language, HSs might experience a delay in late-acquired phenomena but eventually catch up with MSs (Schulz and Grimm, 2019), so we expect, apart from timing, no pronounced qualitative differences between HSs and MSs.

On the discourse level, register awareness creates another source of variation in heritage language use since HSs might not have sufficient exposure to a similarly wide range of registers as MSs of the same language (Polinsky, 2018, pp. 323–324; Aalberse et al., 2019, p. 148). HSs usually experience their heritage language in informal settings, most likely in oral interactions with family members, and might not be as familiar with formal registers. On the other hand, they use their majority language in a greater variety of communicative situations, so they develop a nuanced register awareness comparable to that of MSs of the majority language. Our research has shown that HSs can transfer their register awareness from their majority language to the heritage language, at least while choosing between independent main, coordinate main, and subordinate clauses (Pashkova et al., in press) when all options are available, in principle. What is yet unclear is whether and how this register awareness will manifest itself in a larger speaker sample and within specific sub-domains, such as the use of SC types.

In comparing HSs and MSs in their use of SCs and their types, we will argue that applying the category “native speaker” as a cover term for both these groups obscures a meaningful description of the variation in their patterns of language use. We address this terminological difficulty and propose adding further specification to the category “native speaker,” such as presence of bilingualism, to enhance transparency and replicability. We furthermore briefly explore other variables, such as proficiency, exposure and dominance as potential characteristics for specification.

## Theoretical and Conceptual Background

### The Native Speaker Spectrum

A native speaker has been defined as “a person who learns a language as a child and continues to use it fluently as a dominant language” (Richards and Schmidt, 2013, p. 386). Other characteristics include grammatical and appropriate usage of the native language, self-identification with the community where it is spoken, and intuitions about (un)grammatical structures in that language. Davies (2013) adds creative performance and the ability to translate and interpret into the native language to the list of native speaker characteristics.

However, within these (extra-)linguistic features included in native speaker definitions, only one is uncontroversial and straightforward, namely the childhood acquisition of their L1 (Cook, 1999, p. 187; Davies, 2003, p. 436). Many of the other features mentioned can also be found in L2 speakers: they can use their L2 fluently, grammatically, appropriately, and intuitively, and be creative performers and translators/interpreters. This is the first point of criticism of the category “native speaker”: how helpful is the category to group people with similar patterns of language use if the majority of its defining features appears in non-native speakers’ productions as well (Lowe, 2020, pp. 21–22)?

Beyond linguistic considerations of fluency, accuracy, and intuition, the category “native speaker” has also been criticized for being politically and ideologically charged. It is noted that being a native speaker is associated with power, language ownership, and even positive personality traits (Bonfiglio, 2010). Race, background, and identity play a role in deciding whether a speaker could be a member of the native speaker group. Holliday (2009) writes that a prototypical English native speaker is a white Anglo-Saxon from an English-speaking western country, and those who do not fit this image might be excluded from native speakerhood. Bonfiglio (2010, p. 12) argues that, in some cases, nativeness is judged based on the speaker’s ethnic/immigrant family background and not their language, for instance, Turkish HSs in Germany might not be readily viewed as German native speakers, even though they grew up in Germany and acquired German as one of their L1s.

### Monolinguals and Heritage Speakers on the Native Speaker Spectrum

Monolingual speakers are the least disputed speaker population subsumed under the category “native speaker” as they only acquire their L1 naturalistically. HSs, however, have not always been included in the group of native speakers (Polinsky and Scontras, 2020). On the one hand, this might be surprising because HSs fit the criterion of naturalistic acquisition from early childhood. Some researchers might have excluded HSs from native speakers since they equate nativeness with high proficiency and dominance instead of seeing it as a product of naturalistic L1 acquisition (Kupisch and Rothman, 2018). On the other hand, such a confusion is understandable since we do frequently see differences in HSs’ heritage language productions compared to MSs’. This is, however, an insufficient criterion for excluding HSs from the native speaker continuum as they are not the only group that might differ from a prototypical, highly proficient monolingual native speaker. We also find these differences in MSs with limited experience with the standard language and in late L2 bilinguals who have migrated and shifted dominance to the L2 and are experiencing L1 attrition (Dewaele, 2018; Kupisch and Rothman, 2018).

If the differences between HSs’ and MSs’ productions are not due to HSs being non-native speakers, what could they be attributed to? Many researchers agree that differences in amount and quality of input play a very important role in the eventual outcomes of heritage language acquisition (Montrul, 2016, pp. 117–119; Kupisch and Rothman, 2018; Aalberse et al., 2019, pp. 146–149). These differences in input could lead to variation in heritage language productions, for example, case marking in heritage German (Yager et al., 2015; Zimmer, 2020), inflected infinitives in heritage Brazilian Portuguese (Rothman, 2007), or the encoding of motion events in heritage Turkish (Goschler et al., 2020). However, some areas of the heritage language still display substantial similarity with MSs’ productions, for example, voice onset times in heritage Italian (Nagy, 2015), case morphology in heritage Polish, Russian, and Ukrainian (Łyskawa and Nagy, 2020), or use of classifiers in heritage Cantonese (Nagy and Lo, 2019).

Yet, it would be too simplistic to say that one domain of heritage language grammar and use would show only similarities to MSs’ productions, while another domain would be likely to show only differences. Some areas show both differences and similarities with MSs’ productions. For instance, Brehmer and Usanova (2015) report that verb placement in heritage Russian in Germany is different in SCs compared to monolingual Russian, with an increase in use of the verb in clause-final position, which would be an expected transfer from German. However, main clauses in heritage Russian do not feature more use of the verb in second position (V2, required in German) than those in monolingual Russian. Thus, verb placement in heritage Russian exhibits difference and similarities with monolingual Russian. In a similar vein, our own previous research demonstrated that clause type use across different registers in heritage German also shows a combination of differences and similarities with monolingual German. While independent main clauses are used in the same manner by both speaker groups, coordinate main and subordinate clauses exhibit variation: HSs prefer coordinate main clauses, while MSs choose subordinate clauses more frequently (Pashkova et al., in press).

Concerning HSs’ majority language, their linguistic behavior in everyday interactions is oftentimes comparable to that of MSs, especially once HSs reach early adulthood (Paradis, 2019). For example, HSs have been reported to not have a foreign accent in their majority language (Kupisch et al., 2014). Further, Pashkova et al. (in press) found no evidence that German HSs use different clause type patterns across registers in their majority English, compared to English MSs—overall, both groups used more independent main clauses in the written mode, more coordinate main clauses in the spoken mode, and more subordinate clauses in the formal setting. However, there is experimental evidence that HSs might exhibit more fine-grained differences to English MSs in their majority English, for instance in the release of final stops (Polinsky, 2018, pp. 141–144), grammaticality judgments of subject–verb agreement (Paradis, 2019), and scope assignment (Scontras et al., 2017).

Summing up, HSs are typically native speakers of both of their languages since they typically acquire both languages naturalistically in early childhood. This does not mean, however, that HSs’ linguistic performance is identical to that of prototypical, highly proficient MSs. These two groups of native speakers show differences and similarities in the patterns of their language use. Therefore, we propose further specification of the category “native speaker” in order to reflect this variability. Our study illustrates that an important variable to specify is the presence of bilingualism; additional specifications can include proficiency, exposure, and dominance.

### Subordinate Clauses

The use of SCs and their types across registers is complex in that the speaker requires both syntactic knowledge and register awareness to decide on the appropriateness of SCs according to communicative situations (as explained in section “Register Characteristics of Subordinate Clauses,” SCs are often more preferred in formal contexts). As specified in the Interface Hypothesis, structures involving both syntactic and pragmatic choices are particularly open to variation in terms of acquisition timing and/or cross-linguistic influence (Sorace, 2011; Tsimpli, 2014), thus leading to potentially different patterns across different types of natives speakers. We thereby add subordinate clause choice to the phenomena considered in interface research, given that register is a part of pragmatics, a language-external component (Tsimpli, 2014, p. 301). In the following section, we will examine the syntactic mastery of SCs and register awareness in both speaker groups.

#### Syntactic Characteristics of Subordinate Clauses

##### Subordinate clauses in general

Syntactically, SCs have the following features (Diessel, 2004, p. 48): they are integrated in the matrix clause, they are dependent structures that are formally incomplete without the matrix clause, and they are part of the same processing and planning unit as the associated matrix clause. This last feature is one of the reasons why SCs have been associated with higher syntactic complexity than juxtaposed matrix clauses (Polinsky, 2008; Neary-Sundquist, 2017; Peristeri et al., 2017; Sánchez Abchi and De Mier, 2017; Housen et al., 2019). Syntactic complexity has been defined, among other things, as the extent to which language users resort to syntactic embedding and SCs or as a structure which requires more steps in the syntactic derivation (Housen et al., 2012; Sanfelici and Schulz, 2021). However, the direct link between SCs and syntactic complexity has also been questioned: several researchers reported that textual complexity correlated not with the number of SCs but rather with mean length of nominal phrases and clauses (Lu, 2011; Wiese et al., 2020; Wang and Tao, 2020). Overall, the evidence for high complexity of SCs appears conflicting. Nevertheless, if SCs reflect textual complexity to some extent, we would expect fewer SCs in HSs’ productions in their heritage language compared to MSs of that language.

In addition to the general complexity of SCs across languages, different word order constraints in HSs’ heritage and majority language might play a role in SC production. This study examines HSs of German with English as their majority language. German and English differ in SC word order: In finite clauses introduced by complementizers and relative pronouns, German canonically exhibits subject-object-verb (SOV) structure,^1^ while English has subject-verb-object (SVO) structure. This typological mismatch between the two languages of HSs might make the production of SCs in German harder for HSs than for MSs due to higher cognitive load because of the inhibition of one structure in the bilingual mind—in this case, SVO (Abutalebi and Green, 2016). This may lead to avoidance of SCs in the German productions of HSs (see Pashkova et al., in press, for a more detailed discussion).

##### Subordinate clause types

This section focuses on the syntactic characteristics of SC types and on how they might contribute to the variation between HSs and MSs. We follow previous researchers (e.g., Beaman, 1984; Diessel, 2004; Thompson et al., 2007; Paradis et al., 2017; Andreou and Tsimpli, 2020) in subdividing finite SCs into three categories: complement, adverbial, and relative clauses. In the following, we describe each clause type in detail and provide an overview of their L1 acquisition patterns.

Complement clauses are SCs that function as arguments of a predicate in the matrix clause (e.g., *She saw that a car was coming.*) (Biber et al., 1999, p. 658; Diessel, 2004, p. 1; Noonan, 2007; Lust et al., 2015, p. 301). Some researchers have suggested that complement clauses emerge early in L1 acquisition (Vasilyeva et al., 2008; Paradis et al., 2017), one of the reasons proposed for this being that they are narrowly syntactic structures that only require the knowledge of verb complement selection patterns and no pragmatic skills in discourse management (Mastropavlou and Tsimpli, 2011; Andreou, 2015; Andreou et al., 2020a). In child HSs, the accurate repetition of complement clauses in a sentence repetition task at the ages of 8–12 was reported to be associated with the amount of exposure to the language between ages 0 and 3 and at the age of 6 (Andreou et al., 2020a). This suggests that there are crucial periods for the development of complement clauses that correlate with their production later on. Hence, in the heritage language, we would expect similar production patterns in HSs and MSs because they received similar input at an age when language exposure could affect their emergence.

Adverbial clauses are SCs that modify main clauses similarly to adverbs and adverbial adjuncts modifying a proposition (e.g., *While she was walking, she saw an accident*) (Diessel, 2004, p. 1; Thompson et al., 2007). Contrary to narrowly syntactic structures, adverbial clauses, along with relative clauses, involve the syntax–discourse interface because they rely on discourse and pragmatics and call for discourse management skills (Peristeri et al., 2017, pp. 5, 11; Andreou et al., 2020a, b). For this reason, it has been argued that adverbial clauses are acquired later than complement clauses. Moreover, in child HSs, the accurate repetition of adverbial clauses at the ages of 8–12 was shown to be influenced by current language exposure (Andreou et al., 2020a). This suggests that adverbial clause use might be a locus for greater variation between heritage language and monolingual productions due to differences in the speakers’ current language exposure.

Relative clauses are SCs that modify a noun phrase (NP) (e.g., *A woman who was pushing a baby stroller was walking down the street*) (Andrews, 2007). They are characterized by a syntactic gap that is associated with a relative pronoun at their left periphery and requires as its antecedent the relativized constituent of the matrix clause (Biber et al., 1999, p. 608; Diessel, 2004, p. 117). Similar to adverbial clauses, relative clauses are also located at the syntax-discourse interface and require discourse management skills, i.e., the ability to determine what is needed for referent specification in particular contexts. Therefore, one might expect relative clauses to be more influenced by later exposure, hence leading to greater variation between HSs’ heritage language productions and those of MSs.

In the current study, we investigate whether the suggested differences of the acquisition onset of SC types impacts their use in HSs who are older than those examined in previous research (Andreou et al., 2020a).

#### Register Characteristics of Subordinate Clauses

Register is a variety definable in terms of situational parameters such as participants, channel, purpose, spoken or written mode, and formality of communication (Biber and Conrad, 2001, p. 175). In this study, we operationalize formality as spoken or written communication with public institutions, and informality as spoken or written communication with friends and family. HSs normally do not have as frequent exposure to a variety of registers in their heritage language compared to MSs of that language (Polinsky, 2018, pp. 323–324; Aalberse et al., 2019, p. 148 for recent mention of this tendency). Since the use of the heritage language is mostly limited to interactions with family members and perhaps members of a heritage language community, HSs are usually expected to be more familiar with informal registers and less familiar with formal registers. At the same time, HSs’ majority language typically follows a different trajectory: they use it in a wider range of communicative situations and thus develop formal and informal register repertoires comparable to those of MSs. It is an interesting question, then, how HSs approach formal registers in their heritage language: would they use language patterns from the informal registers of their heritage language or would they try to rely on the formal register patterns from their majority language? Schleppegrell and Colombi (1997) argued for the latter option: they showed that Spanish HSs used very similar clause types in academic essays in heritage Spanish and majority English, despite being unfamiliar with formal academic registers in their heritage language.

Our recent study (Pashkova et al., in press) identified a similar tendency: German HSs showed similar clause type patterns in formal and informal registers in heritage German and majority English, which we called “an underlying register awareness”—HSs were able to transfer their register awareness from their majority language to their heritage language. Crucially, HSs used similar clause type patterns in heritage German compared not only to majority English but also to monolingual German. This possibility of transfer appears viable when the heritage and majority languages have similar register-related language use of the phenomenon under scrutiny, as was the case for clause type use in German and English (in both languages, MSs preferred independent main clauses in the written mode, coordinate main clauses in the spoken mode, and subordinate clauses in the formal setting). It is as yet unclear if register awareness can be attested in a larger data sample and transferred to another phenomenon, such as SC types. However, it is important to note that similar patterns of SC use in heritage and monolingual German did not mean the same frequency of SCs—HSs still used overall fewer SCs than MSs, most likely due to the syntactic characteristics of SCs outlined above.

Subordinate clauses and their types show variation across registers, which makes them an interesting phenomenon to examine with respect to register-related linguistic behavior of HSs. For instance, Koch and Oesterreicher (2012) outlined syntactic features of the language of immediacy, i.e., spontaneous face-to-face dialogues between familiar speakers, and the language of distance, i.e., carefully planned interactions between strangers in the public sphere. The language of immediacy is characterized by parataxis, whereas the language of distance is associated with hypotaxis. Our previous study (Pashkova et al., in press) confirmed this claim: in both English and German, we found more SCs in formal registers, which were similar to the language of distance, than in informal registers, similar to the language of immediacy.

Subordinate clause types are also subject to register variation. In English, for example, Biber and Gray (2016, pp. 87–100) reported more complement and adverbial finite clauses in conversation than in academic writing, and more wh-relative clauses in academic writing than in conversation. Beaman (1984) showed that nominal and relative subordinations occur more often in spoken narratives than in written ones, while adverbial subordinations are more frequent in written productions. Even though these findings do not map directly on the registers examined in the current study (a formal report to the police vs. an informal message to a close friend), we can still expect a certain variation in SC type productions according to formality. Our data will serve as an addition to the research on register repertoires of HSs because, to the best of our knowledge, there has not been a study that focuses on the systematic analysis of SC types according to formality.

#### The Present Study

To address the gaps in the literature just discussed, we pursue the following research questions (RQs) concerning the use of SCs in HSs’ productions. Based on findings from the literature, we also lay out hypotheses and predictions for each question.

**RQ 1**: Do HSs show similarities or differences in the use of SCs according to register in their majority language (English) compared to English MSs and in their heritage language (German) compared to German MSs?

**Hypothesis 1:** Based on our previous study of clause type use in a smaller participant sample (Pashkova et al., in press), we expect HSs to show similarities to English MSs and to differ from German MSs due to syntactic complexity and SOV word order of German SCs.

**Prediction 1:** Comparing HSs’ majority English to monolingual English, we expect to find similar frequencies of SCs in all registers. Comparing HSs’ heritage German to monolingual German, we expect to find similar patterns across registers but overall fewer SCs in heritage German.

**RQ 2**: Do HSs show similarities or differences in the use of SC types (relative, complement, and adverbial) according to formality^2^ in their majority language (English) compared to English MSs and in their heritage language (German) compared to German MSs?

**Hypothesis 2:** We expect HSs to show similarities with English MSs, and a combination of differences and similarities with German MSs due to the different acquisition periods of SC types.

**Prediction 2.1:** Comparing HSs’ majority English to monolingual English, we expect to find similar frequencies of SC types across settings (formal/informal). Comparing HSs’ heritage German to monolingual German, we expect to find similar frequencies of complement clauses but different frequencies of adverbial and relative clauses, since the latter two SC types are assumed to be acquired later than complement clauses.

**Prediction 2.2:** Concerning the heritage language, we also expect to observe larger differences between HSs and MSs in the formal setting since HSs are less familiar with formal registers and we have no previous evidence that they can transfer their register awareness from majority English to heritage German in the use of SC types.

## Materials and Methods

### Participants

For this study we looked at 91 adolescents aged 14–18 years (mean age = 16.1, SD = 1.39, 50 females), with 32 in each of the monolingual groups and 27 in the heritage German group with English as their majority language.

1. HSs of German with majority language English (mean age = 15.6, SD = 1.58, 12 females)
2. MSs of German (mean age = 16.6, SD = 0.91, 19 females)
3. MSs of English (mean age = 16.1, SD = 1.49, 19 females).

The HSs of German grew up speaking German with at least one L1 German-speaking parent in the household (21 HSs had one German-speaking parent, five had two, and one participant provided no answer). All speakers were either born in the United States, or moved there before age two. They did not receive bilingual education, but may have participated in German “Saturday schools” or other German-speaking activities in the community. Speakers of established German “language islands” were excluded from the study. We defined monolinguals as speakers whose L1 was the only language spoken at home, but who might have acquired further languages through foreign language instruction.

German HSs were recruited in Boston, Massachusetts; Madison, Wisconsin; and St. Paul, Minnesota by contacting German organizations and institutions as well as via social media platforms. German MSs were recruited via contacting German high schools in Berlin. English MSs were recruited in the same cities as German HSs (and in Long Island, New York) via social media platforms or through personal contacts. The socio-economic status of HSs’ families was slightly higher than that of English and German MSs (see Supplementary Appendix A for detailed information on parental education) due to the nature of our HS participant pool, which mostly consisted of professionals whose move to the United States was work-related.

The German and English productions of the HSs as well as those of the English MSs were elicited in the United States and those of German MSs in Germany. The data for this study is openly accessible via the Research Unit Emerging Grammars (RUEG) 0.4.0 corpus (Wiese et al., 2020). Both English and German productions of HSs were compared to the productions of MSs of the respective language.

### Materials and Procedure

The data was collected using the Language Situations methodology (RUEG group, 2018; Wiese, 2020), which elicits controlled, comparable, and quasi-naturalistic productions across registers. Participants watched a short non-verbal video depicting a minor car accident and recounted what they saw, imagining themselves witnesses to the accident. The procedure was divided into two settings. In the formal setting, the elicitor was formally dressed and met with the participant in a room set up like an office. In the informal setting, the elicitor was casually dressed and met with the participant in a more relaxed setting, with snacks and beverages offered. In order to enhance an easy-going, comfortable atmosphere, the elicitor and the participant engaged in 10–15 min of task-unrelated conversation in the target language at the beginning of the informal session. The participant watched the video three times in total (twice in the first setting, once in the second setting) and was then asked to recount it in two different modes: spoken and written.

The formal recounting was operationalized as a voice message to a police hotline (spoken) and a witness report to the police (written), while the informal recounting comprised a WhatsApp voice message (spoken) and a WhatsApp text message (written) to a friend. The order of settings (formal/informal) and modes (spoken/written) was balanced across participants. The MSs completed all tasks in one session. The HSs completed the tasks in two sessions—one for their majority language (English) and one for their heritage language (German)—with an interval of 3–5 days in between to minimize priming effects. The order of language sessions was counterbalanced across participants. Upon completion of all the narrative tasks, the participants filled out an online questionnaire^3^ about their language background as well as a self-assessment of their abilities in each language. Self-assessment showed that HSs rated their speaking and writing skills higher in their majority English (speaking mean = 5, SD = 0; writing mean = 4.96, SD = 0.19) than in heritage German (speaking mean = 3.66, SD = 0.78; writing mean = 2.81, SD = 1.27). English monolinguals rated their skills comparably high (speaking mean = 4.75, SD = 0.51; writing mean = 4.53, SD = 0.57) to German monolinguals (speaking mean = 4.96, SD = 0.17; writing mean = 4.66, SD = 0.66).

### Data Coding

As mentioned above, we investigated the use of SCs and their types (complement, adverbial, and relative) in narratives in English and German. In both languages, we examined only clauses that contained finite verbs to constrain the nature of the question. Morphologically non-canonical clauses, i.e., deviations with respect to person and number agreement, were still included, since they do not affect the type that the clause is assigned to. Subordinations missing complementizers or relative pronouns were included because a large proportion of the data stems from spoken productions and omitting complementizer “that” or relative pronouns “who” and “which” (in English) is common in spoken productions (Biber and Conrad, 2001). Non-finite constructions, such as infinitives, present participles, and past participles were excluded. All narratives were split into finite clauses, and each clause was coded for being an SC or a matrix clause. In German, SCs mostly exhibited finite verb-final structures, with the exception of unintroduced complement clauses (see below).^4^ Weil V2 clauses were not coded as SCs since *weil* has lost its status of a subordinator in those constructions (Antomo and Steinbach, 2010; Reis, 2013).

Each SC was coded for its type: complement, adverbial, or relative.^5^ We included both verb and noun complement clauses in our analysis even though the majority of L1 acquisition literature focuses on verb complements. Noun complement clauses usually complement a certain set of nouns such as *question, thought, report*, *argument* (Biber et al., 1999, pp. 645–656), and therefore appeared quite rarely in our data due to the content of the video. Since there were not enough cases to group them into a separate category, they were collapsed with verb complement clauses. Verb complement clauses (1a) should not be confused with what follows multi-word discourse markers *I think, I mean, I don’t know, you know*, which look like epistemic expressions. In order to differentiate a discourse marker from an epistemic expression, a complementizer test was applied: if a complementizer/wh-pronoun was present or could be added after the expression in question, the expression was not taken to be a discourse marker and, hence, the following part was annotated as a complement clause (1b). If a complementizer was absent and could not be added, the expression was taken to be a discourse marker with no complement clause (1c). Each clause in square brackets in (1) was counted as one complement clause.

(1) a. They weren’t looking and then realized [a car was coming_*complement*_] (USbi52FE_fwE)^6^

b. I don’t know [what else happened_*complement*_] (USbi50FD_isE)

c. And then these two cars came by and like I dunno_*discourse marker*_ they came to the intersection and the guy dropped his ball (USmo64FE_isE)

In complement clauses, German exhibits finite verb-final structures (2a), but also allows for canonical V2 structures, if the complementizer is omitted after verbs of saying and thinking (2b). Each clause in square brackets in (2) was counted as one complement clause.

(2) a. und konnte daher nicht wissen [ob nach der Ball ein Mensch kommen würde_*complement*_]

(USbi64MD_fwD)

“And due to this (the driver) could not know if a person would come after the ball.”

b. Ich hoffe [ich konnte ihnen behilflich sein_*complement*_]!^7^ (DEmo54FD_fwD)

“I hope I could be of help to you!”

All types of adverbial clauses (e.g., temporal, locative, causative, conditional, concessive) were put into one category. Each clause in square brackets in (3) was counted as one adverbial clause.

(3) a. I witnessed the crash [as I was walking along the side of a street_*adverbial*_] (USbi55FD_fwE)

b. The car stopped short [because there was a dog trying to get the ball_*adverbial*_] (USmo59FE_iwE)

c. [Als sie die straße überqueren wollten_*adverbial*_], ist der Mann den Ball aus dem Hand gefallen.

(USbi64MD_fwD).

“As they wanted to cross the street, the ball dropped out of the man’s hand.”

As for relative clauses, we included not only those modifying an NP (4a,b) but also those modifying an entire proposition (4c,d) (Biber et al., 1999, p. 867). The reasoning here was similar to the inclusion of noun complement clauses: even though the majority of L1 acquisition literature focuses on NP-modifying relative clauses, there were a few cases of proposition-modifying relative clauses, which were, however, not numerous enough to form their own category, so they were collapsed with NP-modifying relative clauses. Even though there has been extensive research on different types of relative clauses in HSs (e.g., Polinsky, 2011; Albirini and Benmamoun, 2014), we did not distinguish between object and subject relative clauses because we did not have sufficient data points to perform a separate comparison of the two types. Each clause in square brackets in (4) was counted as one relative clause.

(4) a. it tried to like stop for this dog [that was running into the street_*relative*_] (USmo65FE_isE)

b. Ein Mann [der anscheinend mit seiner Frau spazieren war_*relative*_] prellte einen Fußball.

(DEmo69MD_fwD)

“A man who was walking apparently with his wife bounced a soccer ball.”

c. The dog saw the ball and ran for it, [which caused the car in the front to stop_*relative*_].

(USbi51FD_fwE)

d. und is dem ersten auto dann raufgefahren [was zu dem unfall geführt hat_*relative*_]

(DEmo65FD_fsD)

“and drove into the first car which lead to the accident”

Tables 1, 2 show the total number of clause productions in English and German respectively.

**TABLE 1:** English clause productions by speaker group and register/formality.

Register	Formal spoken		Formal written		Informal spoken		Informal written	
Speaker group	HS	MS	HS	MS	HS	MS	HS	MS
All clauses	494	511	424	459	393	430	257	290
Subordinate clauses	145	128	119	144	88	95	58	50

**Formality**	**Formal**				**Informal**			

Speaker group	HS		MS		HS		MS	
Complement clauses	41		49		40		44	
Adverbial clauses	105		114		55		49	
Relative clauses	118		109		51		52	

**TABLE 2:** German clause productions by speaker group and register/formality.

Register	Formal spoken		Formal written			Informal spoken		Informal written	
Speaker group	HS	MS	HS		MS	HS	MS	HS	MS
All clauses	448	732	358		625	370	638	219	399
Subordinate clauses	77	201	90		178	51	114	15	69

**Formality**	**Formal**					**Informal**			

Speaker group	HS			MS		HS		MS	
Complement clauses	74			138		19		53	
Adverbial clauses	22			65		23		69	
Relative clauses	71			176		24		61	

### Data Analysis

First, the data was coded for SCs and matrix clauses, resulting in a dependent variable “Clause type” with two levels (1 for SC and 0 for matrix clause). We analyzed the use of SCs vs. matrix clauses using generalized binomial linear mixed effect models in R (R Core Team, 2021) and the lme4 package (Bates et al., 2015). We maximally specified the fixed effects by including all theoretically relevant independent variables and their interactions: bilingualism (heritage bilingual/monolingual), setting (formal/informal), mode (spoken/written). We contrast-coded the factors using sum contrast coding (–0.5/0.5). We attempted to maximally specify the random effect of participants and included the random slopes for setting and mode (Barr et al., 2013). The maximal specification worked for German SCs, but not for English SCs, where it led to overfitting, so we removed the random slopes and left only the random intercept.

Second, each SC was coded for its type, resulting in a dependent variable “SC type” with three levels (complement, adverbial, and relative). Then, we recoded the dependent variable “SC type” into three separate dependent variables “Complement clause”, “Adverbial clause”, and “Relative clause” with two levels (1 and 0). After this manipulation, each SC type was analyzed independently from the other two types also using generalized binomial linear mixed effect models. Due to the small sample size of each SC type (Tables 1, 2), we collapsed the spoken and written modes within each setting and only included the independent variables of bilingualism (heritage bilingual/monolingual) and setting (formal/informal) and their interaction. We contrast-coded the factors using sum contrast coding (–0.5/0.5). Where possible, we maximally specified the random effect of participants by including the random slopes for setting. If this led to a perfect correlation of fixed effects or a random effect variance estimated at 0 or 1, we removed the random slope. In the next section, we report the *z*- and *p*-values of the models, for full model summaries, see Supplementary Appendix B.

## Results

### Majority and Monolingual English

#### Subordinate Clauses in English

**FIGURE 1:**
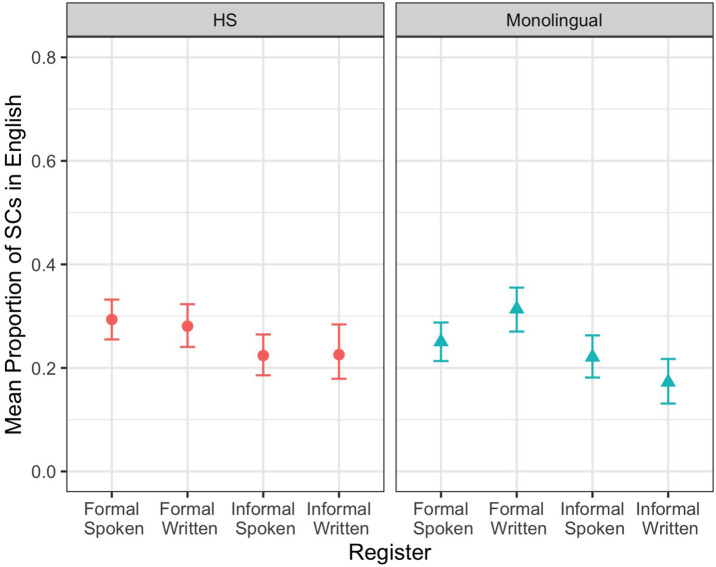
Mean proportion of SCs in English by speaker group and register.

For English SCs, we observed a main effect of setting (*z* = 4.70, *p* ≤ 0.001): speakers produced more SCs in the formal setting more than in the informal setting (Figure 1). In addition, we observed a three-way interaction between bilingualism, setting, and mode (*z* = 2.02, *p* = 0.043). To interpret this interaction, we ran separate models for HSs and MSs. HSs showed a main effect of setting (*z* = 2.71, *p* = 0.007), while MSs showed a main effect of setting (*z* = 4.04, *p* ≤ 0.001) and an interaction between setting and mode (*z* = –2.46, *p* = 0.014). Tukey’s multiple comparison test (MCT, run with *emmeans* package, Lenth, 2021) revealed a significant difference between the formal and the informal settings in the written mode (estimate = –0.51, SE = 0.16, *z* = –3.26, *p* = 0.006) and an absence of such a difference in the spoken mode (estimate = 0.19, SE = 0.16, *z* = 1.24, *p* = 0.602). This shows that German HSs and English MSs partially overlapped in their SC productions. While they behaved similarly in the written mode, they diverged in the spoken mode: HSs distinguished between the settings whereas MSs did not. Additionally, for both speaker groups, setting played a key role in SC production.

#### Subordinate Clause Types in English

**FIGURE 2:**
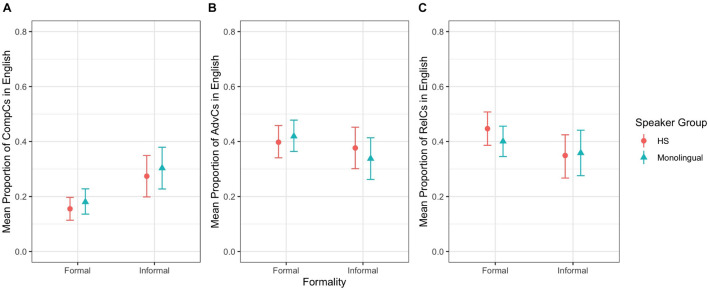
Mean proportion of SC types in English by speaker group and formality: **(A)** Complement Clauses, **(B)** Adverbial Clauses, **(C)** Relative Clauses.

For English complement clauses, we observed a main effect of setting (*z* = –3.73, *p* ≤ 0.001): there were fewer complement clauses in the formal setting than in the informal one (Figure 2A). For English adverbial clauses and relative clauses, we did not observe any main effects or interactions (Figures 2B,C). These results indicate that German HSs and English MSs performed similarly regarding the production of all SC types, and formality played a role only for complement clauses, with fewer complement clauses in the formal setting.

### Heritage and Monolingual German

#### Subordinate Clauses in German

**FIGURE 3:**
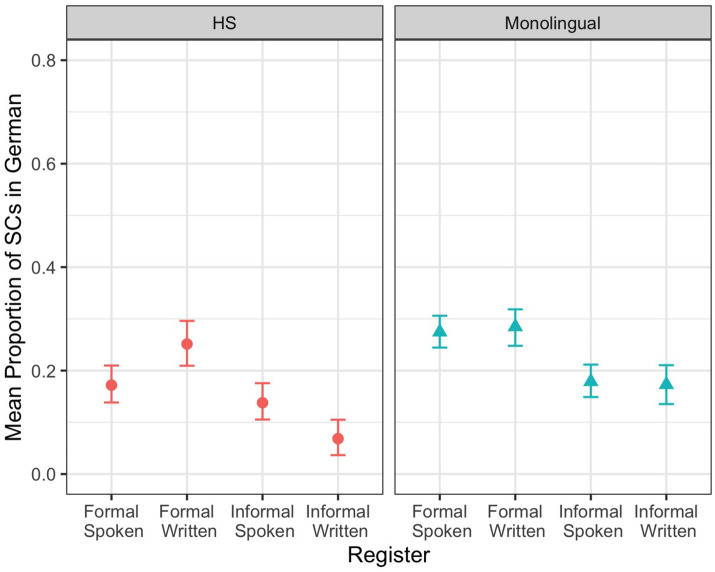
Mean proportion of SCs in German by speaker group and register.

For German SCs, we observed two main effects and two interactions. First, there was a main effect of bilingualism (*z* = –3.55, *p* ≤ 0.001), with HSs producing fewer SCs than MSs (Figure 3). Second, we found a main effect of setting (*z* = 6.35, *p* ≤ 0.001): there were more SCs in the formal setting than in the informal setting. Then, we observed an interaction of setting and mode (*z* = –2.98, *p* = 0.003), with a greater difference between the formal and informal settings in the written mode (estimate = 1.08, SE = 0.18, *z* = 5.94, *p* ≤ 0.001) than in the spoken mode (estimate = 0.45, SE = 0.13, *z* = 3.37, *p* = 0.004), according to Tukey’s MCT. Finally, we observed a three-way interaction between bilingualism, setting, and mode. To interpret it, we ran separate models for HSs and MSs. The HS model indicated a main effect of setting (*z* = 4.61, *p* ≤ 0.001), with more SCs in the formal setting than in the informal setting. In addition, there was an interaction of setting and mode. Tukey’s MCT revealed a difference between the formal and informal setting in the written mode (estimate = 1.45, SE = 0.30, *z* = 4.84, *p* ≤ 0.001) but not in the spoken mode (estimate = 0.22, SE = 0.20, *z* = 1.09, *p* = 0.698). The MS model showed only a main effect of setting (*z* = 4.36, *p* ≤ 0.001). This shows that German HSs and MSs differed in the overall SC productions: while HSs distinguished between the settings only in the written mode, MSs did so in both modes. In addition, for both speaker groups, setting played a key role in SC production.

#### Subordinate Clause Types in German

**FIGURE 4:**
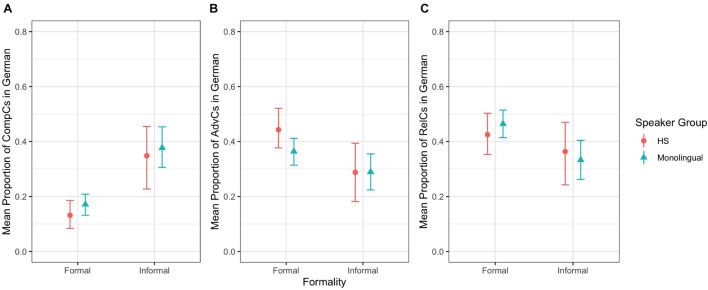
Mean proportion of SC types in German by speaker group and formality: **(A)** Complement Clauses, **(B)** Adverbial Clauses, **(C)** Relative Clauses.

For German complement clauses, we observed a main effect of setting (*z* = –5.74, *p* ≤ 0.001), with fewer complement clauses in the formal setting than in the informal setting (Figure 4A). For adverbial clauses, we observed a main effect of setting (*z* = 2.90, *p* = 0.004), with more adverbial clauses in the formal setting than the informal setting (Figure 4B). For relative clauses, we observed a main effect of setting (*z* = 2.30, *p* = 0.022), with more relative clauses in the formal setting than the informal setting (Figure 4C). These results indicate that German HSs and German MSs performed similarly regarding the production of all SC types. Formality played a role for both speaker groups: they produced fewer complement clauses but more adverbial clauses and relative clauses in the formal setting than the informal setting.

## Discussion

This study aimed at presenting reasons for why the category “native speaker” is flawed and should be further specified to account for the variation between the groups that fall under its scope. Such a specification would enhance transparency and replicability of research. We analyzed two native speaker groups—HSs and MSs—to argue that there are differences and similarities, as well as a combination of both, between the groups. In particular, we compared German HSs residing in the United States with English and German MSs. We looked at the use of SCs and their types (complement, adverbial, and relative) in spoken and written narratives across registers.

Our first research question focused on whether HSs use finite SCs in a similar or different way in their majority language compared to English MSs and in their heritage language compared to German MSs. With respect to HSs’ majority language, our data does not confirm Hypothesis 1 and Prediction 1, which state that in their majority language, German HSs will perform similarly to English MSs. Overall, both speaker groups produce more SCs in the formal setting, confirming previous results, thus exhibiting similarity (see Pashkova et al., in press). This similarity is however only partial because a closer look at SC productions across registers reveals that HSs distinguished between the settings in spoken and written modes while MSs did so only in the written mode. With respect to HSs’ heritage language, our data confirms Hypothesis 1 and Prediction 1, which state that in their heritage language, German HSs will produce significantly fewer SCs than German MSs. Additionally, HSs distinguished between the settings only in the written mode, while MSs did so in both modes. This can be attributed to the cognitive load of spoken online productions in combination with the general complexity of SCs and word order differences in SCs in English and German (Pashkova et al., in press).

Our second research question zoomed in on the use of finite SC types according to formality. We wanted to know whether HSs would show similarities or differences in their majority language compared to English MSs and in their heritage language compared to German MSs. With respect to HSs’ majority language, our data confirms Hypothesis 2 and Prediction 2.1, which state that HSs and MSs should show similar frequencies of SC types across settings. With respect to HSs’ heritage language, our data does not confirm Hypothesis 2 and Prediction 2.1, which expect a combination of differences and similarities between HSs and MSs, because both speaker groups in fact behaved similarly regarding the frequencies of SC types across settings. Consequently, we did not find any support for Prediction 2.2, which argued for a bigger difference between HSs and MSs in the formal setting.

Overall, the results show that the locus of variation between HSs and MSs is not where we predicted it to be. For English SCs, we expected to find only similarities between HSs and MSs, and instead we observed a combination of differences and similarities. HSs adhere to formality distinctions regardless of mode, unlike English MSs, who do so only in the written mode. This could be attributed to the different attitudes toward our study among HSs and MSs: HSs were well aware that their language competence was under scrutiny, and were probably trying to show their best language skills. This is especially true for the heritage language but could also have influenced their performance in the majority language, which might explain their strict adherence to the formality distinction in both modes. This illustrates that the two groups of native speakers show variation in their performance, potentially due to extralinguistic factors such as their perception of the situation. Therefore, the category “native speaker” groups together speakers with different patterns of language use and is not specific enough to allow comparability in a speaker population.

Another unpredicted result is that in German, HSs behave similarly to MSs with regard to all SC types, even adverbial and relative clauses, which we expected to differ between the speaker groups due to their later acquisition and location at the syntax-discourse interface. This is contrary to the previous findings by Andreou et al. (2020a), who showed that the current language exposure influences the production of adverbial clauses by child HSs in a sentence repetition task. However, their participants were much younger than ours (mean age 9.01 vs. mean age 15.6), which could be the reason for the discrepancy in our results. Perhaps, the use of adverbial clauses is influenced not only by the current language exposure but also by the overall cognitive maturity of the speaker (see Paradis et al., 2017 on the advantages of higher cognitive maturity in early L2 acquisition). Furthermore, the absence of difference could be attributed to the relatively small sample size in this study, which could have prevented us from capturing it. Productions of more speakers need to be analyzed to confirm our result. The analysis of SC types and SCs in German illustrated that we can still find similarities within a narrower phenomenon (SC types) between the sub-groups of native speakers even if a more general phenomenon (SCs) shows differences between the same speaker groups.

An additional unexpected finding was that concerning SC types, HSs behaved similarly to German MSs in their heritage language and similarly to English MSs in their majority language, even though the MSs of the respective languages behaved differently—in English, formality only had an effect on complement clauses, whereas in German, formality had an effect on all SC types. This shows that German and English differ in their formality-related language use and that HSs are able to adapt to the MS pattern in both their languages. This is surprising since the HSs’ ability to adjust their SC type productions in their heritage language does not appear to originate from their exposure to formal registers in German or from transfer of their formality awareness from English into German. Further research is needed to pinpoint the source of this behavior.

The presented findings lead us to the conclusion that the category “native speaker” is too general to adequately define a speaker population because the speakers subsumed under this category may well differ in their linguistic behavior. Therefore, we argue for a more specific categorization, which provides more fine-grained information on their language background, allowing the possibility of capturing both group and individual variation, which are gradient (Ortega, 2020). Previous literature suggests that the category” “native speaker” should be replaced with “L1 user” (Dewaele, 2018). We argue for the necessity of further specification since even within L1 users, we can see differences as illustrated throughout this paper. This specification could include information on bilingualism, language exposure, proficiency, and dominance. In the current statistical analysis, we included only the variable of bilingualism in heritage language context. Further studies are needed to examine the influence of proficiency, language exposure, and dominance, which we expect to play a role in the variability among native speakers. Following this suggestion, for example, the majority of our German HSs could be described as bilinguals who are simultaneously raised in German and English, residing in the United States, with English as their current dominant language and German as their less dominant language. A typical German MS could be described as a monolingually-raised German speaker, residing in Germany, with German as their current dominant language.

One limitation of the present study, as already mentioned, is the relatively small sample size of the three SC types, which did not allow us to look into the interaction of bilingualism, formality and spoken/written mode. Since this interaction proved significant in the SC use, it would be very interesting to examine it in SC types as well. Due to a small sample size, we also were not able to assess potential qualitative differences in SC types (word order, choice of complementizer, or verb placement). Another possible extension of the current study is to examine further heritage-majority language pairs, probably typologically more distant, to see whether the patterns we describe here would manifest themselves in other native speaker groups. The RUEG corpus, which provided the data analyzed in this study, is a useful resource for such an extension since it contains comparable data for Greek, Turkish, and Russian HSs in Germany and the United States, plus data for their monolingual counterparts. Another aspect that could be addressed in future studies is the register-related language use in English, German, and possibly other languages. It is noteworthy that English and German MSs in our study did not behave similarly with respect to formality, and further research would be needed to uncover the possible sources of this difference. An additional step could be the inclusion of a wider range of registers with the same formality and mode distinctions, to see whether the formality sensitivity is tied to a particular situation (e.g., a police report) or if it is more general.

## Conclusion

This study investigated the appropriateness of the category “native speaker” by comparing productions of two native speaker groups, namely heritage and monolingual speakers. We assessed the use of SCs and their types (complement, adverbial, and relative) in narratives produced by adolescent HSs of German in the United States in comparison with adolescent German and English MSs. We provided evidence that there are similarities, differences, and a combination of both in the productions of HSs and MSs. Our results show similarities in the production of SC types between HSs’ majority English and monolingual English, as well as between heritage and monolingual German. Differences were found in SC productions between heritage and monolingual German. A combination of differences and similarities was found in SC productions between majority and monolingual English. These findings support existing criticism of the category “native speaker” and further highlight its underspecification. As is, the category fails to adequately reflect the variation among speaker groups who fall under its scope. Therefore, we argue that we should enhance the category “native speaker” with more specific descriptions of speaker groups in order to provide unambiguous information about them.

## Data Availability Statement

The datasets presented in this study can be found in online repositories. The names of the repository/repositories and accession number(s) can be found below: RUEG corpus https://doi.org/10.5281/zenodo.3236068.

## Ethics Statement

The studies involving human participants were reviewed and approved by the Deutsche Gesellschaft für Sprachwissenschaft ethics committee and the Institutional Review Board (IRB) University of Maryland at College-Park. Written informed consent to participate in this study was provided by the participants’ legal guardian/next of kin.

## Author Contributions

All authors listed have made a substantial, direct and intellectual contribution to the work, and approved it for publication.

## Conflict of Interest

The authors declare that the research was conducted in the absence of any commercial or financial relationships that could be construed as a potential conflict of interest.

## Publisher’s Note

All claims expressed in this article are solely those of the authors and do not necessarily represent those of their affiliated organizations, or those of the publisher, the editors and the reviewers. Any product that may be evaluated in this article, or claim that may be made by its manufacturer, is not guaranteed or endorsed by the publisher.

## Footnotes

1 Unintroduced subordinate clauses require verb-raising into second position, as in main clauses. Those cases are also accounted for in this study, see section “Data Coding.”

2 Due to the small sample size of SC types, we decided to collapse the four registers (formal spoken, formal written, informal spoken, informal written) into two formality conditions—formal vs. informal.

3 Questionnaire for adolescent participants of the Research Unit Emerging Grammars; https://osf.io/qhupg/

4 We also included seven non-canonical V2 clauses clearly conceptualized as SCs: three complement clauses, two adverbial clauses, two relative clauses. We did not conduct a separate analysis V2 SCs due to their low frequency.

5 We did not conduct fine-grained qualitative analyses of SC types such as examining word order, choice of complementizers or verb placement, although these characteristics are definitely worth exploring in further research. We did so since any further subdivision on the data would result in a too low number of data points in each subcategory to conduct a statistical analysis.

6 The participant code in the examples includes the following information: US/DE, country of elicitation, United States or Germany; bi/mo, bilingual/monolingual speaker; 01, speaker number; M/F, speaker’s sex; D/E, HS’s heritage language (Deutsch for German) or monolinguals’ L1 (English or German); f/i, formal/informal setting; s/w, spoken/written mode; D/E, language of elicitation, D for German or E for English.

7 We preserved the original orthography of the written productions.
