# Supplementary material for: Deconstructing the Native Speaker: Further Evidence From Heritage Speakers for Why This Horse Should Be Dead!
Source: Front Psychol. 2021 Oct 5;12:717352. doi: 10.3389/fpsyg.2021.717352 (PMC8523891; doi:10.3389/fpsyg.2021.717352)
Supplement: Supplementary file 1 [file Data_Sheet_1.pdf]

Appendices

Appendix A: Parental education
German HSs

	Mother's education	%	Father's education	%
High school	1	3.5%	0	0%
Associate’s degree	1	3.5%	1	3.7%
Bachelor’s degree	5	17.9%	3	11.1%
**Master’s degree**	12	**43%**	13	**48.1%**
**Doctorate degree**	6	**21.5%**	7	**26%**
College degree (unspecified)	1	3.5%	1	3.7%
NA	2	7.1%	2	7.4%
Total	28	100%	27	100%

Note: Here and in the two tables below the parent counts do not always equal the number of participants in a given speaker group. If the parent count is greater than the participant number it is due to some participants reporting having a stepmother or a stepfather, who were included in the mother or father counts. If the parent count is smaller than the participant count, it is due to the participant not reporting living with their mother/father.
In the current table, the counts include one stepmother and one stepfather.

English monolinguals

	Mother's education	%	Father's education	%
High school	2	6.1%	6	19.3%
Associate’s degree	4	12.1%	2	6.5%
**Bachelor’s degree**	12	**36.4%**	7	**22.6%**
**Master’s degree**	8	**24.2%**	9	**29%**
Doctorate degree	3	9.1%	3	9.7%
College degree (unspecified)	1	3%	1	3.2%
N/A	3	9.1%	3	9.7%
Total	33	100%	31	100%

Note: The counts include two stepmothers and two stepfathers.

German monolinguals

	Mother's education	%	Father's education	%
**High school (mittlere Reife / mittlerer Schulabschluss (MSA) / 10. Klasse)**	4	14.8%	2	11.1%
High school (Abitur / Fachabitur)	5	**18.5%**	3	16.7%
Vocational training (abgeschlossene	5	**18.5%**	4	22.2%

Berufsausbildung / Fachschulabschluss)				
**College degree ((Fach)Hochschulabschluss)**	12	**44.5%**	9	**50%**
N/A	1	3.7%	0	0%
Total	27	100%	18	100%

Note: The counts include two stepfathers.

Appendix B: Model tables

Table 1: Generalized linear mixed effects model (GLMM) English SCs

Model parameter	Estimate	SE	z	p
(Intercept)	-1.157	0.056	-20.808	< .001
Bilingualism	0.121	0.111	1.088	.277
Setting	0.405	0.086	4.699	< .001
Mode	0.004	0.086	0.045	.964
Bilingualism:Setting	-0.166	0.173	-0.964	.335
Bilingualism:Mode	0.030	0.172	0.177	.860
Setting:Mode	-0.259	0.172	-1.510	.131
Bilingualism:Setting:Mode	0.694	0.343	2.020	.043

Model formula: e_sc_model_no_slopes <- glmer(sc ~ biling_sum*setting_sum*mode_sum+(1|speaker), data=e, family = binomial, control = glmerControl(calc.derivs=FALSE))
The model with random slopes had a singular fit, so we took the slopes out.

Table 2: GLMM English SCs, HSs only

Model parameter	Estimate	SE	z	p
(Intercept)	-1.076	0.062	-17.400	< .001
Setting	0.327	0.121	2.706	.007
Mode	0.026	0.121	0.212	.832
Setting:Mode	0.074	0.242	0.308	.758

Model formula: e_sc_HS_model <- glmer(sc ~ setting_sum*mode_sum+(1|speaker), data=e_HS, family = binomial, control = glmerControl(calc.derivs=FALSE))

Table 3: GLMM English SCs, MSs only

Model parameter	Estimate	SE	z	p
(Intercept)	-1.237	0.090	-13.731	< .001
Setting	0.495	0.122	4.044	< .001
Mode	-0.013	0.122	-0.110	.912
Setting:Mode	-0.601	0.244	-2.465	.014

Model formula: e_sc_mono_model <- glmer(sc ~ setting_sum*mode_sum+(1|speaker), data=e_mono, family = binomial, control = glmerControl(calc.derivs=FALSE))

Table 4: Tukey's multiple comparison test, English SCs by setting and mode, MSs only

Contrast	Estimate	SE	df	z	p
formal spoken - informal spoken	0.194	0.157	Inf	1.240	.602
formal spoken - formal written	-0.314	0.145	Inf	-2.164	.133
formal spoken - formal written	0.481	0.188	Inf	2.554	.052
informal spoken - formal written	-0.508	0.156	Inf	-3.258	.006

informal spoken - informal written	0.287	0.197	Inf	1.461	.461
formal written - informal written	0.796	0.187	Inf	4.243	< .001

Pairwise comparison formula: pairs(lsmeans(e_sc_mono_model, ~setting_sum*mode_sum, adjust="tuckey"))

Table 5: GLMM English CompCs

Model parameter	Estimate	SE	z	p
(Intercept)	-1.449	0.122	-11.843	< .001
Bilingualism	-0.165	0.245	-0.672	.501
Setting	-0.682	0.183	-3.733	< .001
Bilingualism:Setting	-0.129	0.366	-0.354	.724

Model formula: e_comp_model <- glmer(comp ~ biling_sum*setting_sum+(1+setting|speaker), data=e %>% drop_na(sc_type), family = binomial, control = glmerControl(calc.derivs=FALSE))

Table 6: GLMM English AdvCs

Model parameter	Estimate	SE	z	p
(Intercept)	-0.481	0.080	-6.019	< .001
Bilingualism	0.038	0.160	0.239	.811
Setting	0.220	0.151	1.449	.147
Bilingualism:Setting	-0.255	0.303	-0.841	.400

Model formula: e_adv_model <- glmer(adv ~ biling_sum*setting_sum+(1+setting|speaker), data=e %>% drop_na(sc_type), family = binomial, control = glmerControl(calc.derivs=FALSE))

Table 7: GLMM English RelCs

Model parameter	Estimate	SE	z	p
(Intercept)	-0.419	0.100	-4.177	< .001
Bilingualism	0.061	0.200	0.302	.763
Setting	0.283	0.163	1.735	.083
Bilingualism:Setting	0.302	0.326	0.926	.355

Model formula: e_rel_model <- glmer(rel ~ biling_sum*setting_sum+(1+setting|speaker), data=e %>% drop_na(sc_type), family = binomial, control = glmerControl(calc.derivs=FALSE))

Table 8: GLMM German SCs

Model parameter	Estimate	SE	z	p
(Intercept)	-1.640	0.086	-19.085	< .001
Bilingualism	-0.611	0.172	-3.554	< .001
Setting	0.767	0.121	6.353	< .001
Mode	0.110	0.108	1.017	.309
Bilingualism:Setting	0.283	0.241	1.173	.241
Bilingualism:Mode	0.187	0.216	0.868	.386
Setting:Mode	-0.626	0.210	-2.983	.003
Bilingualism:Setting:Mode	-1.164	0.420	-2.772	.006

Model formula: g_sc_model <- glmer(sc ~ biling_sum*setting_sum*mode_sum+(1+setting+mode|speaker), data=g, family = binomial, control = glmerControl(calc.derivs=FALSE))

Table 9: Tukey's multiple comparison test, German SCs by setting and mode

Contrast	Estimate	SE	df	z	p

formal spoken - informal spoken	0.453	0.135	Inf	3.370	.004
formal spoken - formal written	-0.203	0.111	Inf	-1.829	.259
formal spoken - formal written	0.876	0.179	Inf	4.908	< .001
informal spoken - formal written	-0.657	0.143	Inf	-4.591	< .001
informal spoken - informal written	0.423	0.181	Inf	2.330	.091
formal written - informal written	1.080	0.182	Inf	5.939	< .001

Pairwise comparison formula: pairs(lsmeans(g_sc_model, ~setting_sum*mode_sum, adjust="tuckey"))

Table 10: GLMM German SCs, HSs only

Model parameter	Estimate	SE	z	p
(Intercept)	-1.939	0.146	-13.309	< .001
Setting	0.834	0.181	4.610	< .001
Mode	0.153	0.180	0.848	.397
Setting:Mode	-1.232	0.360	-3.426	.001

Model formula: g_sc_HS_model <- glmer(sc ~ setting_sum*mode_sum+(1|speaker), data=g_HS, family = binomial, control = glmerControl(calc.derivs=FALSE))
The model with random slopes had a singular fit, so we took the slopes out.

Table 11: Tukey's multiple comparison test, German SCs by setting and mode, HSs only

Contrast	Estimate	SE	df	z	p
formal spoken - informal spoken	0.218	0.201	Inf	1.086	.698
formal spoken - formal written	-0.463	0.179	Inf	-2.594	.047
formal spoken - formal written	0.987	0.302	Inf	3.273	.006
informal spoken - formal written	-0.682	0.199	Inf	-3.431	.003
informal spoken - informal written	0.769	0.312	Inf	2.460	.066
formal written - informal written	1.450	0.300	Inf	4.840	< .001

Pairwise comparison formula: pairs(lsmeans(g_sc_HS_model, ~setting_sum*mode_sum, adjust="tuckey"))

Table 12: GLMM German SCs, MSs only

Model parameter	Estimate	SE	z	p
(Intercept)	-1.324	0.095	-14.007	< .001
Setting	0.595	0.136	4.362	< .001
Mode	0.018	0.111	0.158	.874
Setting:Mode	-0.039	0.212	-0.183	.855

Model formula: g_sc_mono_model <- glmer(sc ~ setting_sum*mode_sum+(1+setting+mode|speaker), data=g_mono, family = binomial, control = glmerControl(calc.derivs=FALSE))

Table 13: GLMM German CompCs

Model parameter	Estimate	SE	z	p
(Intercept)	-1.249	0.136	-9.165	< .001
Bilingualism	-0.290	0.273	-1.062	.288
Setting	-1.333	0.232	-5.742	< .001
Bilingualism:Setting	-0.267	0.464	-0.576	.565

Model formula: g_comp_model <- glmer(comp ~ biling_sum*setting_sum+(1+setting|speaker), data=g %>% drop_na(sc_type), family = binomial, control = glmerControl(calc.derivs=FALSE))

Table 14: GLMM German AdvCs

Model parameter	Estimate	SE	z	p
(Intercept)	-0.664	0.106	-6.289	< .001
Bilingualism	0.188	0.211	0.888	.374
Setting	0.545	0.188	2.904	.004
Bilingualism:Setting	0.385	0.376	1.024	.306

Model formula: g_adv_model <- glmer(adv ~ biling_sum*setting_sum+(1+setting|speaker), data=g %>% drop_na(sc_type), family = binomial, control = glmerControl(calc.derivs=FALSE))

Table 15: GLMM German RelCs

Model parameter	Estimate	SE	z	p
(Intercept)	-0.509	0.136	-3.742	< .001
Bilingualism	-0.042	0.272	-0.155	.877
Setting	0.480	0.209	2.298	.022
Bilingualism:Setting	-0.338	0.418	-0.808	.419

Model formula: g_rel_model <- glmer(rel ~ biling_sum*setting_sum+(1+setting|speaker), data=g %>% drop_na(sc_type), family = binomial, control = glmerControl(calc.derivs=FALSE)
